# A QSAR Study Based on SVM for the Compound of Hydroxyl Benzoic Esters

**DOI:** 10.1155/2017/4914272

**Published:** 2017-07-03

**Authors:** Li Wen, Qing Li, Wei Li, Qiao Cai, Yong-Ming Cai

**Affiliations:** ^1^School of Public Health, Guangdong Pharmaceutical University, Guangzhou Higher Education Mega Centre, Guangzhou 510006, China; ^2^Guangdong Provincial Center for Disease Control and Prevention, Guangzhou 511430, China; ^3^School of Medical Information Engineering, Guangdong Pharmaceutical University, Guangzhou Higher Education Mega Centre, Guangzhou 510006, China

## Abstract

Hydroxyl benzoic esters are preservative, being widely used in food, medicine, and cosmetics. To explore the relationship between the molecular structure and antibacterial activity of these compounds and predict the compounds with similar structures, Quantitative Structure-Activity Relationship (QSAR) models of 25 kinds of hydroxyl benzoic esters with the quantum chemical parameters and molecular connectivity indexes are built based on support vector machine (SVM) by using R language. The External Standard Deviation Error of Prediction (SDEP_ext_), fitting correlation coefficient (*R*^2^), and leave-one-out cross-validation (*Q*^2^_LOO_) are used to value the reliability, stability, and predictive ability of models. The results show that *R*^2^ and *Q*^2^_LOO_ of 4 kinds of nonlinear models are more than 0.6 and SDEP_ext_ is 0.213, 0.222, 0.189, and 0.218, respectively. Compared with the multiple linear regression (MLR) model (*R*^2^ = 0.421, RSD = 0.260), the correlation coefficient and the standard deviation are both better than MLR. The reliability, stability, robustness, and external predictive ability of models are good, particularly of the model of linear kernel function and eps-regression type. This model can predict the antimicrobial activity of the compounds with similar structure in the applicability domain.

## 1. Introduction

### 1.1. Conceptual Framework

QSAR [1, 2] is used to research the relationship between the molecular structure and biological activity and physicochemical characteristics, reveal the quantitative relationship, predict the activity of unknown compounds, and direct the synthesis of new materials [3–5]. QSAR is considered as one of the promising technologies and is widely used at present because of making up the loss of experimental data, reducing the cost of testing, and achieving high throughput prediction and screening [6]. Many international organizations and regulatory agencies have supported and promoted the use of QSAR and thought that QSAR can be used as an alternative to animal experiments. Health Canada, the United States of Food and Drug Administration (FDA), Environmental Protection Agency (EPA), the European Union, and the Organization for Economic Cooperation and Development (OECD) apply QSAR to identify potential health hazards, screening, and priority [7]. After recent years of development, QSAR has become a frontier topic in medicinal chemistry, environmental chemistry, life science, analytical chemistry, computer chemistry, and even pesticide [8–11].

Hydroxyl benzoic esters are important kinds of preservatives, which are widely used in medicine, food, cosmetics, pesticides, and other fields [12]. At present, there are about 60 kinds of food preservatives in the world [13]. The benzoic acid and sorbic acid are productive in China, but the usage is little because of the high toxicity of benzoic acid and the high price of sorbic acid. Hydroxyl benzoic esters have high efficiency, low toxicity, compatibility, and other advantages; the performance of antibacterial is stronger than benzoic acid and sorbic acid because it has a phenolic hydroxyl [14]. So it is of great significance to study and apply the antibacterial activity of hydroxyl benzoic esters.

### 1.2. Research Status of SVM in QSAR

SVM is a machine learning algorithm based on statistical learning theory proposed by Cortes et al. [15–17]. SVM can be used for pattern recognition, regression analysis and function fitting, and so forth because it possesses favorable mathematical properties, such as the uniqueness of the solution, nondependence on the dimension of the input space, and so forth. The optimal solution of SVM is superior to the traditional learning methods. In recent years, SVM is applied to the study of QSAR of the compound. Hou et al. [18] investigated the QSAR of the antimalarial activity of PfDHODH inhibitors by generating four computational models using a multiple linear regression (MLR) and a SVM based on a dataset of 255 PfDHODH inhibitors. Sharma et al. [19] drew support from SVM and MLR studying the activity of HIV-1 capsid inhibitors. SVM model was found more efficient in prediction. Khuntwal et al. [20] used MLR and SVM to develop QSAR models for a dataset of 34 tetrahydrobenzothiophene derivatives. Zhiming et al. [21] by using ridge regression (RR) and SVM built QSAR models of bitter tasting thresholds (BTT) and cytotoxic T lymphocyte (CTL) and predicted independent test data. Results showed that the fitting, LOOCV, and external prediction accuracies were superior to the reported results of the existing literature. Zhang et al. [22] took the benzene compounds as the research object, combining the molecular structure of the quantitative description with MLR or nonlinear regression statistical methods SVM, to build successfully the acute toxicity QSAR models and mutagenic QSAR models of benzene compounds. By comparing the linear and nonlinear QSAR models, Zhang Xiao-Long discovered that the stability and prediction ability of nonlinear QSAR models are better than those of multiple linear QSAR models. In the literature, there are very few researches about QSAR of the hydroxyl benzoic esters. Jiang et al. [23] used MLR to build the model of QSAR and it can well predict the MIC and* t*_0.5_ in the range of atomic number (the number of C among 1–4 on the ester chain of MIC and 1–3 of *t*_0.5_). Qiu et al. [24] optimized the molecular structures of eleven kinds of p-hydroxyl benzoic esters by using density functional theory (DFT) B3LYP method of quantum chemistry and then used stepwise multiple linear regression to select the descriptors and to generate the best prediction model that relates the structural features to inhibitory activity. The QSAR results showed that the lowest unoccupied molecular orbit *E*_LUMO_ and the increase of dipole moment *μ* were the main independent factors contributing to the antifungal activity of the compounds. SVM has shown obvious advantages in the QSAR research, but QSAR study of the compound of hydroxyl benzoic esters is confined to the linear model at present; there is no literature on the nonlinear QSAR analysis of the system.

In this paper, we use the quantum chemical parameters and molecular connectivity indexes to analyze the antibacterial activity of the hydroxyl benzoic esters. The QSAR model is established by the SVM algorithm in the R software. We obtain the structure-activity relationship between the molecular structural parameters and the antibacterial activity of* Escherichia coli* under the most stable configuration, which provides a basis of predicting the antibacterial activity of similar compounds.

## 2. Method

### 2.1. Data Preparation

#### 2.1.1. Basic Information of Hydroxyl Benzoic Esters

This paper took the 25 hydroxyl benzoate group compounds as the research object, including 10 o-hydroxyl benzoic esters, 2 m-hydroxyl benzoic esters, and 13 p-hydroxyl benzoic esters. Their details are shown in Table 1.

#### 2.1.2. Terminal Value

The antimicrobial half-life (*t*_1/2_) (h) at the condition of minimum inhibition concentration of 25 hydroxyl benzoic esters was collected from the literature [23], in the form of logarithm (lgt_1/2_) to express its antibacterial activity. The results are shown in Table 2.

### 2.2. Calculation and Selection of Molecular Descriptors

The quantum chemical parameters [25] and molecular connectivity indexes [26] can well explain the antibacterial activity of compounds and have good correlation between them; therefore, this paper selects them with a clear physical meaning as the descriptor.

#### 2.2.1. The Quantum Chemical Parameters

In this paper, the quantum chemical parameters are calculated by the latest Gaussian 09 software [27] that is a quantum chemistry software of semiempirical calculation and ab initio calculation of United States Gaussian company. Gaussian 09 in the calculation can carry out the molecular structure through the View Gauss 5 software directly and create the input files of molecular structures. In the calculation, Gaussian 09 software calls directly the input file and translates it into the form of redundant internal coordinates automatically. The results of the calculation are output by the text. Each time before calculation, a suitable chemistry model (computational method) should be established for the system in order to achieve balance in terms of computational cost and accuracy [27, 28]. The method of this paper is B3LYP/6-31G DFT/(d). Because all the molecular configurations are optimal configurations and the geometry optimization is convergent and there is no virtual frequency by the frequency analysis, therefore, all the data are true and reliable. Find out the useful quantum chemical parameters from the output file. The values are shown in Table 3.

#### 2.2.2. The Molecular Connectivity Indexes

Molecular connectivity indexes which mainly reflect the number of atoms in molecules, valence bond and branch information, and so forth are the constants that are calculated according to the molecular structure. Each order index has a different meaning. Many studies show that ^5^**X**^**v**^_**P**_ can characterize a lot of information, which has a great significance in explaining the influence of structure on biological activity [29, 30]. So, this study selects 8 molecular connectivity indexes, including ^0^**X**^**v**^_**P**_, ^1^**X**^**v**^_**P**_, ^2^**X**^**v**^_**P**_, ^3^**X**^**v**^_**P**_, ^4^**X**^**v**^_**P**_, ^5^**X**^**v**^_**P**_, ^3^**X**^**v**^_**C**_, and ^4^**X**^**v**^_**PC**_. The results are shown in Table 4.

### 2.3. Establishment of Models

#### 2.3.1. Partition of Dataset

The rational division of datasets is a very hot research topic in the field of QSAR. There are a variety of methods. In this paper, Random Sampling (RS) [31] is used to divide the raw data into training set (22 kinds) and test set (3 kinds, o-hydroxyl benzoic esters, m-hydroxyl benzoic esters, and p-hydroxyl benzoic esters). The training set is used to establish the SVM nonlinear models, and the test set tests the external prediction ability of the models.

#### 2.3.2. Modeling Method

Through the R software program, the training set with 22 compounds is used to build the nonlinear models by SVM algorithm based on the selected descriptors. Firstly, we standardize the data and then establish 4 models of kernel for radial, linear, eps-regression, and nu-regression type, respectively.

### 2.4. Model Validation

Model validation is very important for QSAR research, which consists of two aspects: internal validation to test the fitting ability and robustness of models and external validation to test the model's predictive ability. Both internal and external validations are equally important [32].

#### 2.4.1. Internal Validation

There are many methods to estimate a model's stability, robustness, and internal predictive ability, such as the fitting correlation coefficient, cross-validation, random model test, Y random, and various residual errors (like Root Mean Squared Errors (RMSEs), standard residual error, etc.) [33]. In this paper, the fitting correlation coefficient (*R*^2^) between the experimental and predicted values of the training dataset and leave-one-out cross-validation (*Q*^2^_LOO_) are used to test the reliability, robustness, stability, and whether the models are overfitting or not.

#### 2.4.2. External Validation

A very important purpose of the QSAR models is to predict the related activity data of new or even nonsynthetic compounds, in order to guide the design and synthesis of compounds with desirable activity, or to screen the compounds. This requires that the model has good predictive ability and generalization ability; however, cross-validation can only explain the internal predictive ability of models and good internal prediction ability does not mean the excellent external prediction ability [34–36]; that is, good cross-validation *Q*^2^_cv_ is a necessary but nonsufficient condition for the high external predictive ability [35]. The only way to evaluate the external predictive ability of the model is to test the model with the new compound (namely, external test set that is not involved in the process of descriptor selection and model establishment). The parameters of evaluation model's external predictive ability include *R*^2^_ext_, external *Q*^2^_ext_, and SDEP_ext_. In this paper, the test set is used to predict the corresponding lgt_1/2_ and external predictive ability of the models is evaluated by SDEP_ext_.

### 2.5. Extraction of Key Descriptors

We use principal component analysis to extract the most critical molecular descriptors of the hydroxyl benzoic esters for antibacterial half-life.

## 3. Results

### 3.1. Internal Prediction and Scatter Plot

**Figure 1:**
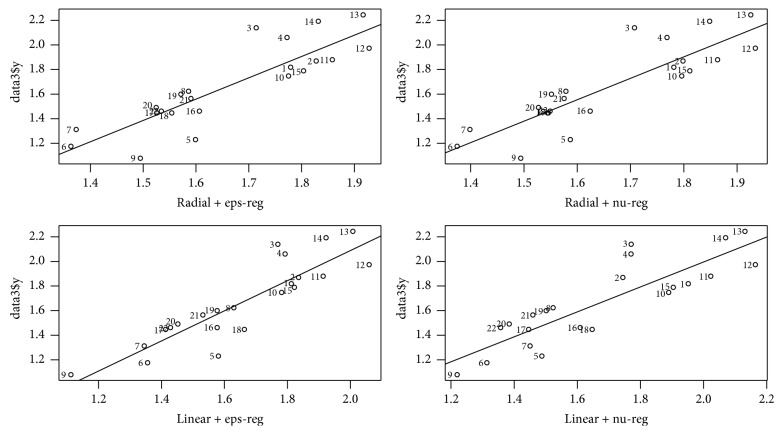
Scatter plot of experimental values and 4 SVM models' internal prediction results of lgt1/2.* Note*. The horizontal coordinates, respectively, represent the predicted values of lgt1/2 of 4 SVM models, and the longitudinal coordinates express the experimental results.

Four nonlinear SVM models based on the selected descriptors are established by using training set. Experimental values and internal prediction results of lgt_1/2_ are shown in Table 5 and scatter plot in Figure 1.

### 3.2. Parameters of Internal Validation

 See Table 6.

### 3.3. Results of External Validation

**Figure 2:**
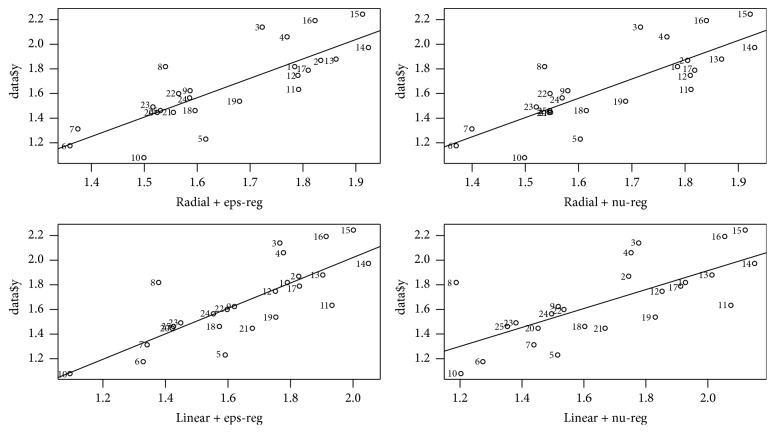
Scatter plot of experimental values and 4 SVM models' prediction results of lgt1/2.* Note*. The horizontal coordinates, respectively, represent the predicted values of lgt1/2 of 4 SVM models, and the longitudinal coordinates express the experimental results.

lgt_1/2_ of the test set is predicted, respectively, by 4 SVM models and the results are shown in Table 7. SDEP_ext_ of the models and the residual between experimental values and the predicted results of lgt_1/2_ are displayed in Table 8. Scatter plots of experimental values and prediction results by 4 SVM models of 25 compounds of lgt_1/2_ are shown in Figure 2.

### 3.4. Results of Principal Component Analysis

 See Tables 10 and 11.

## 4. Discussion and Conclusion

The degree of freedom and the speed of the preservative molecule determine the effective collision between the central atom of reactivity and the group or atom of microbial molecular activity. As a result, the antimicrobial property of the preservative is essentially determined by the electronic behavior of the preservative and the microorganism, that is, the quantum biochemical characterization of preservative. Therefore, from the perspective of quantum chemistry to study the relationship between the structure and properties of compound, the effective antimicrobial groups of preservative can be explained in essence [37]. Jiang et al. [23] use multiple linear regression to establish the linear model of 25 kinds of hydroxyl benzoic esters. The parameters are shown in Table 9. Results showed that *R*^2^ was only 0.421, but the equation had good linear relationship when the number of C atoms was less than 4. When the number of C atoms in the ester group is more than 4, the influencing factors become more complex and cannot be described by simple linear relationship and may be in nonlinear or diversified relationship. So we use the R language to write the program and establish 4 kinds of nonlinear models through the SVM machine algorithm for 25 hydroxyl benzoic esters and predict lgt_1/2_. Predicted results of training set are shown in Table 5. The scatter plot of experimental and predicted lgt_1/2_ is drawn by using R software. Figure 1 shows that the predicted and experimental values are in good agreement and the linearity is obvious. According to literatures, if the value of *R*^2^ is greater than 0.6 [35, 38] and *Q*^2^ is greater than 0.5, the model is good, and model is excellent when the values are more than 0.9 [39]. Tropsha et al. [6] recommend *R*^2^ and *Q*^2^ to be greater than 0.6. Table 6 shows that both *R*^2^ and *Q*^2^_LOO_ are greater than 0.6 and *R*^2^ and *Q*^2^_LOO_ of two models with linear kernel function are close to 0.75, so we may think that the stability, robustness, and internal predicted ability of the 4 models are better and the models are not overfitting because *R*^2^ is larger than *Q*^2^_LOO_ by no more than 25%. By RS extracting, the para-, ortho-, and metacompound from 25 hydroxyl benzoic esters make up external test set to test the models, and the prediction results are shown in Table 7. The parameters from Table 8 show that the residual values of lgt_1/2_ of the test set are in the range of −0.037244~0.322733 and SDEP_ext_ is 0.213, 0.222, 0.189, and 0.218, respectively. The results indicate that the 4 models have high external predictive ability among themselves; in particular the model of the linear kernel function and eps-regression type is better than the other 3 models. Scatter plots of experimental values and prediction results by 4 SVM models of 25 compounds of lgt_1/2_ are shown in Figure 2. The results show that the overall prediction of the 4 SVM models is better and, particularly, the linear relationship between predictive and experimental value of the model, where kernel function is linear and type is eps-regression, is the best.

In Table 10, the principal component analysis shows that the proportion of variance of the first principal component reaches 96.03%; therefore, the first principal component is taken only.  Table 11 shows that the first principal component includes *E* (total energy), ZPE (zero-point vibrational energy), and *p* (polarizability). We consider that *E*, ZPE, and *p* are the key factors for antibacterial half-life of hydroxyl benzoic esters. *p* is a kind of structural parameter characterized by molecular deformation tensor under the action of external electric field. It is the most important property that *p* is related to the volume of the molecule and *p* contains information about the molecular interaction that is able to characterize the properties of the molecule as an electron acceptor. Since the coefficients of *p* and ZPE are negative, this indicates that the value of *p* and ZPE is greater and the antibacterial half-life of hydroxyl benzoic esters is shorter but E is just the opposite because the coefficient is positive.

In summary, QSAR nonlinear model obtained by quantum chemical parameters and molecular connectivity indexes can better predict the antibacterial activity of hydroxyl benzoic esters. The introduction of SVM algorithm solves the problem of poor correlation of QSAR and complex nonlinear relationship between the molecular descriptors when formula weight is large, which provides a basis for the prediction of the antibacterial activity of compounds with similar structure.

Therefore, the main conclusions of this paper are as follows:

1. The establishment of the 4 kinds of nonlinear models using 25 hydroxyl benzoic acid esters by SVM method, through internal and external validation, the stability, and robustness, and internal and external predictive ability of 4 kinds of models are good; that is, the models are available and may predict new compounds in the applicability domain.
2. The model of linear kernel function and eps-regression type has the largest *R*^2^ and *Q*^2^_LOO_, the minimum SDEP_ext_, and the optimal linear relationship between predictive and experimental value of lgt_1/2_ in 4 kinds of SVM models, which is the optimal model.
3. SVM algorithm is a good method to solve the problem of multicollinearity and complex nonlinear relationship between molecular descriptors in QSAR modeling.
4. E, ZPE, and p are the key factors for antibacterial half-life of hydroxyl benzoic esters.

## Figures and Tables

**Table 1:** The basic information of hydroxyl benzoate esters.

ID	Compound	Abbreviation
1	Methyl o-hydroxyl benzoate esters	M-o-HB
2	Ethyl o-hydroxyl benzoate esters	E-o-HB
3	Propyl o-hydroxyl benzoate esters	P-o-HB
4	Isopropyl o-hydroxyl benzoate esters	IP-o-HB
5	Butyl o-hydroxyl benzoate esters	B-o-HB
6	Isobutyl o-hydroxyl benzoate esters	IB-o-HB
7	Isoamyl o-hydroxyl benzoate esters	IA-o-HB
8	Octyl o-hydroxyl benzoate esters	O-o-HB
9	Benzyl o-hydroxyl benzoate esters	Be-o-HB
10	Phenyl o-hydroxyl benzoate esters	Ph-o-HB
11	Methyl m-hydroxyl benzoate esters	M-m-HB
12	Ethyl m-hydroxyl benzoate esters	E-m-HB
13	Methyl p-hydroxyl benzoate esters	M-p-HB
14	Ethyl p-hydroxyl benzoate esters	E-p-HB
15	Propyl p-hydroxyl benzoate esters	P-p-HB
16	Isopropyl p-hydroxyl benzoate esters	IP-p-HB
17	Butyl p-hydroxyl benzoate esters	B-p-HB
18	Isobutyl p-hydroxyl benzoate esters	IB-p-HB
19	Amyl p-hydroxyl benzoate esters	A-p-HB
20	Isoamyl p-hydroxyl benzoate esters	IA-p-HB
21	Heptyl p-hydroxyl benzoate esters	H-p-HB
22	Octyl p-hydroxyl benzoate esters	O-p-HB
23	Isooctyl p-hydroxyl benzoate esters	IO-p-HB
24	Nonyl p-hydroxyl benzoate esters	N-p-HB
25	Benzyl p-hydroxyl benzoate esters	Be-p-HB

**Table 2:** Antimicrobial half-life (*t*_1/2_) (h) at the condition of minimum inhibition concentration of hydroxyl benzoic esters.

ID	*t*_1/2_	lgt_1/2_
1	66.0	1.819
2	74.0	1.869
3	138.0	2.139
4	115.0	2.060
5	17.0	1.230
6	15.0	1.176
7	20.5	1.312
8	66.0	1.819
9	42.0	1.623
10	12.0	1.079
11	43.0	1.633
12	56.0	1.748
13	76.0	1.880
14	94.0	1.973
15	176.0	2.245
16	156.0	2.193
17	61.5	1.789
18	29.0	1.462
19	34.5	1.537
20	28.0	1.447
21	28.0	1.447
22	39.8	1.599
23	31.0	1.491
24	36.8	1.565
25	29.0	1.462

**Table 3:** Quantum chemical parameters of hydroxyl benzoic group at B3LYP/6-31G(d) level.

ID	*E*	ZPE	*E*_HOMO_	*E*_LUMO_	Δ*E*	*μ* (*T*_*D*_)	*μ* (*D*_*Y*_)	*p*
	(hartree)	(KJ/mol)	(e V)	(e V)	(e V)	*μ* (*D*_*X*_)	*μ* (*D*_*Z*_)	
1	−535.4	390.3	−0.2384	−0.06313	0.17524	2.736	0.964	105.3
						2.560	0.000	
2	−574.7	464.7	−0.2370	−0.06142	0.17557	3.026	−1.464	117.8
						−2.648	0.000	
3	−614.0	539.2	−0.2367	−0.06093	0.17573	3.155	−1.440	130.0
						2.808	0.000	
4	−614.0	537.7	−0.2359	−0.05991	0.17596	3.091	1.167	130.7
						−2.862	0.000	
5	−653.3	614.2	−0.2364	−0.06066	0.17576	3.182	−2.906	142.3
						1.294	0.000	
6	−653.3	613.0	−0.2368	−0.06100	0.17578	3.169	2.434	142.2
						−1.990	−0.393	
7	−692.6	688.3	−0.2363	−0.06060	0.17574	3.165	2.334	154.6
						2.136	0.047	
8	−810.6	912.9	−0.2362	−0.06035	0.17580	3.276	−2.377	191.1
						2.254	0.000	
9	−766.4	603.6	−0.2381	−0.05969	0.17842	1.302	−0.182	165.9
						−1.093	0.755	
10	−727.1	526.4	−0.2421	−0.06882	0.17325	0.922	0.755	165.9
						0.529	0.000	
11	−574.7	461.5	−0.2408	−0.05736	0.18346	3.350	3.297	94.45
						0.594	0.000	
12	−535.4	387.3	−0.2419	−0.05896	0.18293	3.284	3.053	84.22
						−1.208	0.0006	
13	−535.4	387.8	−0.2450	−0.05006	0.19497	1.278	−1.247	106.0
						0.280	0.000	
14	−574.7	462.1	−0.2439	−0.04860	0.19528	1.194	−0.169	118.4
						−1.182	0.000	
15	−614.0	537.0	−0.2436	−0.04820	0.19540	1.201	0.235	130.6
						−1.178	0.000	
16	−614.0	535.2	−0.2433	−0.04748	0.19577	1.054	0.319	131.3
						1.004	0.000	
17	−653.3	611.3	−0.2434	−0.04798	0.19545	1.122	−0.995	142.9
						−0.519	0.000	
18	−653.3	610.6	−0.2439	−0.04838	0.19550	1.288	−1.012	142.8
						−0.785	−0.131	
19	−692.6	686.3	−0.2433	−0.04789	0.19544	1.1777	−0.8744	155.2
						0.7889	0.0000	
20	−692.6	685.6	−0.2434	−0.04795	0.19547	1.086	−0.912	155.2
						−0.589	0.023	
21	−771.3	836.0	−0.2433	−0.04777	0.19550	1.169	−0.179	179.6
						−1.155	0.000	
22	−810.6	911.2	−0.2432	−0.04772	0.19552	1.089	−1.058	191.7
						−0.258	0.000	
23	−810.6	910.2	−0.2433	−0.04780	0.19550	1.201	1.113	191.7
						0.414	−0.179	
24	−849.9	984.9	−0.2432	−0.04770	0.19553	1.169	−0.522	203.9
						1.046	0.000	
25	−776.4	601.9	−0.2442	−0.04966	0.19452	1.260	−0.697	174.3
						−0.916	−0.512	

**Table 4:** Molecular connectivity indexes of hydroxyl benzoic group.

ID	^0^ **X** ^**v**^_**P**_	^1^ **X** ^**v**^_**P**_	^2^ **X** ^**v**^_**P**_	^3^ **X** ^**v**^_**P**_	^4^ **X** ^**v**^_**P**_	^5^ **X** ^**v**^_**P**_	^3^ **X** ^**v**^_**C**_	^4^ **X** ^**v**^_**PC**_
1	6.073	3.117	2.009	1.226	0.608	0.259	0.190	0.235
2	6.780	3.705	2.238	1.316	0.706	0.311	0.190	0.223
3	7.487	4.205	2.653	1.478	0.770	0.381	0.190	0.223
4	7.650	4.100	2.971	1.436	0.775	0.349	0.426	0.389
5	8.194	4.705	3.007	1.772	0.885	0.426	0.190	0.223
6	8.358	4.561	3.509	1.588	0.818	0.421	0.599	0.629
7	9.065	5.061	3.836	1.969	0.963	0.415	0.599	0.512
8	11.020	6.705	4.421	2.772	1.623	0.904	0.190	0.223
9	9.167	5.262	3.290	2.157	1.187	0.607	0.308	0.407
10	8.460	4.824	3.014	1.898	1.008	0.425	0.258	0.327
11	6.073	3.111	2.042	1.087	0.647	0.255	0.213	0.321
12	6.780	3.699	2.271	1.177	0.780	0.320	0.213	0.309
13	6.073	3.111	2.038	0.904	0.412	0.173	0.213	0.292
14	6.780	3.699	2.267	0.994	0.508	0.237	0.213	0.280
15	7.487	4.199	2.683	1.155	0.572	0.305	0.213	0.280
16	7.650	4.094	3.001	1.114	0.576	0.281	0.449	0.392
17	8.194	4.699	3.036	1.449	0.686	0.350	0.213	0.280
18	8.358	4.555	3.539	1.266	0.620	0.353	0.622	0.446
19	8.902	5.199	3.390	1.699	0.894	0.431	0.213	0.280
20	9.065	5.055	3.865	1.646	0.765	0.384	0.622	0.568
21	10.310	6.199	4.097	2.199	1.248	0.703	0.213	0.280
22	11.020	6.699	4.451	2.449	1.425	0.828	0.213	0.280
23	11.180	6.555	4.637	2.173	1.374	0.792	0.622	0.568
24	11.730	7.199	4.804	2.699	1.601	0.953	0.213	0.280
25	9.1670	5.256	3.609	1.792	0.823	0.467	0.331	0.464

**Table 5:** Experimental values and 4 SVM models' internal prediction results of lgt1/2.

ID	lgt_1/2_	Radial + eps-reg	Radial + nu-reg	Linear + eps-reg	Linear + nu-reg
1	1.819	1.779216	1.781302	1.812285	1.951165
2	1.869	1.827756	1.798580	1.835602	1.744102
3	2.139	1.713926	1.707563	1.769820	1.770619
4	2.060	1.772371	1.768500	1.792442	1.770267
5	1.230	1.599024	1.587027	1.580694	1.487888
6	1.176	1.363148	1.374539	1.355817	1.313377
7	1.312	1.373201	1.398522	1.345323	1.450414
9	1.623	1.585821	1.578657	1.629972	1.523181
10	1.079	1.494724	1.494087	1.112267	1.219324
12	1.748	1.775344	1.796207	1.781397	1.889308
13	1.880	1.858219	1.863383	1.913434	2.022421
14	1.973	1.927762	1.934197	2.059377	2.163645
15	2.245	1.916552	1.925729	2.008084	2.130922
16	2.193	1.831546	1.848498	1.922720	2.069817
17	1.789	1.803731	1.811289	1.822127	1.903807
18	1.462	1.606335	1.624347	1.576873	1.609259
20	1.447	1.525688	1.545616	1.413420	1.445952
21	1.447	1.554127	1.544298	1.663491	1.646921
22	1.599	1.571473	1.551537	1.576665	1.501430
23	1.491	1.524709	1.527338	1.452021	1.384236
24	1.565	1.590526	1.575234	1.531546	1.459150
25	1.462	1.534525	1.549003	1.428528	1.357148

*Note*. Radial + eps-reg, radial + nu-reg, linear + eps-reg, and linear + nu-reg, respectively, represent the 4 SVM models where kernel function is radial and linear and type is eps-regression and nu-regression.

**Table 6:** *R* ^2^ and *Q*^2^_LOO_ of 4 SVM models.

Parameters	Radial + eps-reg	Radial + nu-reg	Linear + eps-reg	Linear + nu-reg
*R* ^2^	0.614	0.613	0.756	0.740
*Q* ^2^_LOO_	0.611	0.608	0.747	0.731

*Note*. Radial + eps-reg, radial + nu-reg, linear + eps-reg, and linear + nu-reg, respectively, represent the 4 SVM models where kernel function is radial and linear and type is eps-regression and nu-regression.

**Table 7:** Experimental values and prediction results of test set of lgt_1/2_.

ID	8	11	19
Compound	O-o-HB	M-m-HB	A-p-HB
lgt_1/2_	1.819000	1.633000	1.537000
Radial + eps-reg	1.501886	1.762810	1.675167
Radial + nu-reg	1.496267	1.776929	1.691083
Linear + eps-reg	1.559133	1.670244	1.732065
Linear + nu-reg	1.535459	1.711716	1.772964

**Table 8:** Experimental and predicted values of lgt_1/2_ residual and SDEP_ext_.

ID	8	11	19	SDEP_ext_
Radial + eps-reg	0.317114	−0.129810	−0.138167	0.213
Radial + nu-reg	0.322733	−0.143929	−0.154083	0.222
Linear + eps-reg	0.259867	−0.037244	−0.195065	0.189
Linear + nu-reg	0.283541	−0.078716	−0.235964	0.218

**Table 9:** The parameters of the MLR model established by Wang Deng-Ju.

Method	*n*	*R*	*R* ^2^	*R*_ad_	RSD
MLR	25	0.649	0.421	0.311	0.260
MLR	8 (*C* < 4)	0.969	0.938	0.856	0.076

**Table 10:** Contribution rate of the first three principal components.

	Comp. 1	Comp. 2	Comp. 3
Standard deviation	188.7974802	38.08956667	4.3318149940
Proportion of variance	0.9602859	0.03908591	0.0005055312
Cumulative proportion	0.9602859	0.99937176	0.9998772898

**Table 11:** Loadings of the first three principal components.

	Comp. 1	Comp. 2	Comp. 3
*E*	0.467	0.838	0.277
ZPE	−0.871	0.491	
*μ* (*D*_*Y*_)			−0.152
*p*	−0.152	−0.238	0.944
